# Purchase intention for second-hand luxury goods: An empirical study of Chinese consumers

**DOI:** 10.1371/journal.pone.0304967

**Published:** 2024-06-05

**Authors:** Guanfeng Yan, Yanjie Li, Tianhai Zhang, Chenglin Mu

**Affiliations:** 1 School of Engineering, Sichuan Normal University, Chengdu, P.R. China; 2 Department of Arts, Science, and Technology, Sichuan Normal University, Chengdu, P.R. China; Federal University of Goias: Universidade Federal de Goias, BRAZIL

## Abstract

Second-hand luxury goods feature both characteristics of luxury products like perceived value including social, emotional, and quality value, and second-hand goods like price-performance ratio. Enlarging the second-hand luxury market is of significance to protect the environment and save rare and valuable natural resources, and thus investigating the determinants of purchase intention is meaningful. From the perspective of the psychology of consumers, the influence of factors related to consumers (recycling awareness, subjective norms, attitudes, perceived behavioral control) and products (perceived value, price-performance ratio) on the intention to buy second-hand luxury goods is explored in this study through an online survey with Chinese consumers as a sample. The results are analyzed using the structural equation model (SEM) and show that consumers’ attitudes, perceived behavioral control, and recycling awareness will promote the intention of purchasing second-hand luxury goods, and the perceived value and price-performance ratio of second-hand luxury goods also have a positive impact on the purchase intention. However, there is no significant relationship between subjective norms and purchase intention. In addition, this study also explores the interrelationship between constructs and draws corresponding conclusions, providing references for the subsequent development of the second-hand luxury market.

## Introduction

With the development of the global economy, the global luxury market is expanding while China has a high share of luxury consumption. Meanwhile, due to the globalization of luxury goods, more and more luxury goods are accepted and desired by the public, which is the reason for the further expansion of the luxury market [1]. With the increase in people’s income, the luxury market may be considered a developing mass market, which includes not only members of the richest social class but also those at a modest income level [2]. There are two main reasons for this phenomenon: firstly, an increasing number of consumers want to emulate the lifestyles of the wealthy by consuming products and services of higher quality [3,4]; secondly, some consumers buy them to satisfy their hedonism. From the perspective of economic development, the luxury market is conducive to promoting global economic development [5]. A clear understanding of the factors that influence consumers to buy luxury goods is necessary for the marketing of luxury brands.

According to previous studies in the business field, many factors affect consumers’ willingness to buy luxury goods. The unique demand of consumers is one of the most frequently mentioned influencing factors, and means that consumers want to pursue individual characteristics and hope to convey an individual identity that can be separated from others. The raw materials used in luxury goods are relatively precious, and the appearance design of the goods is also unique. In addition, the price of luxury goods is much higher than that of common goods while the quantity of luxury goods is small, which is recognized by people as a symbol of uniqueness and caters to consumers’ desire for uniqueness [6]. In addition, the influence of consumers’ self-monitoring ability on their intention to buy luxury goods is also one of the concerns of many studies. Such self-monitoring ability usually refers to consumers’ sensitivity to others’ evaluation [7]. That is, people with high self-monitoring will adjust their behavior in time according to the evaluation of people around them, so they will be more inclined to buy luxury goods that can exhibit their good image [8]. The social function of luxury goods and consumers’ emotional attitudes also affect consumers’ willingness to buy luxury goods to a large extent. As luxury brands convey high prestige and an upscale image, consumers will buy luxury goods to elevate their social reputation [9–11]. Especially under the influence of Chinese collectivism, the signal sent by luxury goods accords with the mentality of consumers to a great extent, which can improve the social image of consumers and encourage them to buy luxury goods [12]. It is undeniable that many consumers will buy luxury goods to satisfy their hedonic values, making their emotional attitudes tilted towards buying luxury goods. To sum up, the social function of luxury goods as well as consumers’ unique needs, self-monitoring ability, and emotional attitude are important factors affecting consumers’ willingness to buy luxury goods.

With the further development of society, the awareness of environmental protection is gradually enhanced thanks to education and media. Especially for luxury goods whose raw materials are rare and precious, the goods are generally of high quality and can be resold as second-hand luxuries to save rare natural resources and preserve the ecological environment [13]. Generally, second-hand goods are cheaper than brand-new goods, and so are second-hand luxuries, which means that consumers would obtain similar commodity quality while pay less money. Consequently, a previous study has shown that the price-performance ratio of second-hand luxury goods is also an incentive to consumers [14]. Therefore, the second-hand luxury market emerges conforming to the trend of the times. Apart from the price-performance ratio, recycling awareness may also encourage consumers to buy second-hand goods. Considering that reducing resource waste is one of the advantages of second-hand luxury goods [15], and protecting limited natural resources is always an important task for us. Under the influence of numerous environmental protection policies, people’s awareness of recycling will be enhanced, and they will be inclined to buy products with higher environmental benefits. Second-hand luxury goods can meet the psychological needs of consumers to protect the environment, so the relationship between recycling awareness and second-hand luxury purchase intention is worth further investigation. Consequently, the purchase of second-hand luxuries can not only meet consumers’ demand for luxury goods but also improve the utilization rate of rare and valuable natural resources. Through the cross-research of different disciplines, more favorable suggestions could be put forward for the win-win situation of the environment and economy.

The luxury market expanded with the increase in the income level of society due to continuous global economic development. Even so, buying new luxury goods is still difficult for most people, so consumers with relatively low incomes choose to buy second-hand luxury goods. There are many examples of second-hand luxury goods purchased out of thrift mentality, so second-hand luxury goods are increasingly favored by moderate and low consumers who account for a large proportion of the whole population [16]. Therefore, several scholars pay attention to the second-hand luxury market, and much research on the potential reasons for second-hand luxury purchases is carried out accordingly. A previous study [17] shows that the value of second-hand luxury goods is mainly reflected in environmental protection and the value of the commodity itself. A survey shows that second-hand luxury goods still have the social value and brand benefits they represent [18]. Moreover, the resale of second-hand luxury goods in the second-hand market proves that it has withstood the test of time, the basic performance of the product has not been affected, and its quality value still exists [18]. So, even if second-hand luxuries are worn to varying degrees after a period of use, their existence is still very meaningful. In terms of second-hand luxury purchases, most previous studies have focused on people’s attitudes, subjective norms, price-performance ratio, perceived behavioral control, and perceived value. Among them, perceived behavioral control and subjective norms are the main contents of goal behavior theory, which provides the basis for explaining the motivation and ability of individuals [19]. However, when it comes to the perceived value of second-hand luxury goods, the quality value of goods is rarely studied while most studies focus on emotional value and social value [14].

In this study, a questionnaire was used to explore the impact of attitude, perceived value, recycling awareness, price-performance ratio, subjective norms, and perceived behavioral control on the intention to buy second-hand luxury goods. The perceived value is divided into three aspects: social value, emotional value, and quality value. Compared with new luxury goods, second-hand luxury goods have a higher price-performance ratio and more significant environmental benefits. Therefore, this study focuses on the analysis of consumers’ awareness of recycling and the price-performance ratio and seeks a green and sustainable development direction to protect the environment while developing the economy. Although existing studies have delineated some of the factors influencing consumers’ purchase of second-hand luxury goods [20], the complexity of consumer behavior and the interdependence of factors driving luxury consumption are rarely acknowledged. In addition to analyzing the influence of the above six factors on the intention to buy second-hand luxury goods, this study will also analyze the interrelationship between several important factors, aiming to promote the long-term development of the second-hand luxury market.

## Literature review

### Perceived value

**Fig 1:**
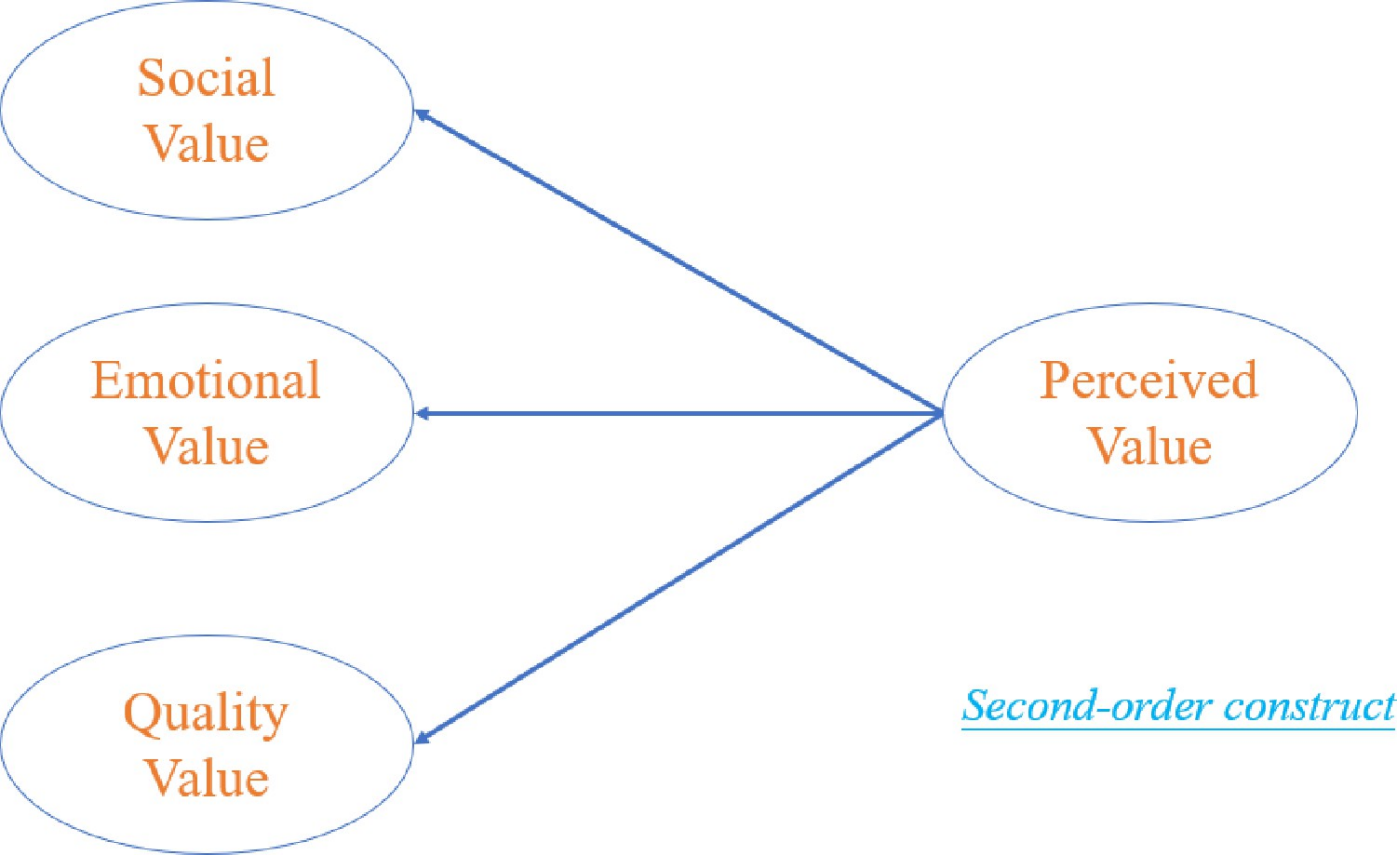
Second-order construct—Perceived value.

Research shows that perceived value is often a significant topic in marketing research [21,22], which refers to consumers’ subjective value perception of products or services [23]. Tu et al. propose the theory of perceived value for the first time, which holds that consumers’ purchase choices depend on the values presented by the products [24]. Therefore, the perceived value of second-hand luxury goods is one of the important incentives that drive consumers to buy such products [25]. Most of the previous studies focused on the social value, personal value, functional value, and property value of second-hand luxury goods [26–29], but less on the quality value and emotional value. However, some consumers still pay high attention to quality value and their emotional value because consumers regard these two aspects of value as something they can directly see, touch, and feel, which are important principles for judging a product. So, shopping experience and product performance are also important to consumers and influence their decisions. This study will discuss the perceived value of second-hand luxury goods from three aspects including social value, emotional value, and quality value, which makes perceived value a second-order construct shown in Fig 1.

#### Social value

The social value of second-hand luxury goods is mainly reflected in the social value brought by luxury brands. As luxury brands themselves have symbolic meanings and signals of social stratification, the main motivation of luxury consumption is to meet social needs rather than basic material needs [30]. This social demand is mainly embodied in integrating the symbolic meaning of luxury brands into the identity of consumers, seeking social recognition, leaving a good social impression, and thus improving the social status of consumers [31]. For example, consumer vanity compels the consumption of display-oriented luxury brands and services [32]. In the age of materialism, the improvement of social status will undoubtedly bring us a lot of conveniences. For example, when attending certain occasions, people with high social status are often given special treatment, so this is one of the reasons why consumers pursue luxury brands. When we see people who use luxury goods, we automatically associate them with high social status and economic power. In this way, the impression of these people in society will be significantly improved and they will be recognized by the people around them to a greater extent. Studies have shown that the sense of superiority brought by luxury brands and people’s purchase intentions are mutually promoting [14]. This sense of superiority will further motivate consumers to pay higher prices for functionally equivalent goods with more social value, as they aspire to gain higher social status through this display of wealth. Although luxury goods laws were gradually abolished after the 18th century, the practice of using personal items as a symbol of social status markers still exists, so social value is an important dimension of perceived value.

#### Emotional value

In terms of emotional value, consumers with hedonistic motivation buy luxury goods to satisfy their emotional value [33]. This emotional value is mainly reflected in two aspects. On the one hand, it can experience personal rewards through consumption, which is manifested as joy when receiving compliments from others. On the other hand, it can improve their inner satisfaction due to the exquisite commodity itself. In addition to this basic need for hedonism, consumers experience the positive emotions and joy associated with treasure hunting when they find limited edition or iconic items in the second-hand market, which are not available in the primary market anymore. In this case, second-hand luxury goods also may be a rarity and can satisfy consumers’ desire to be different from the people around them. What’s more, Kessous et al.’s research found that compared with brand-new luxuries, some second-hand luxuries express the emotions of the original owners of the goods. If the consumer knows the story related to the commodity or previous owner, a personalized relationship can be established between the product and the consumer [23]. Moreover, some iconic items from the past will remind consumers of events of a certain era and evoke feelings of nostalgia. The satisfaction brought by buying second-hand luxury goods is highly consistent with the hedonic value, so this emotional value will encourage consumers to buy second-hand luxury goods.

#### Quality value

Perceived quality is defined as consumers’ judgment of the overall excellence of a product, and the quality of the product itself is an important factor in determining whether consumers will buy it [34]. This represents what the product itself is, not the brand behind it [35]. The social value and emotional value mentioned above are mainly analyzed from the perspective of luxury brands to explore the perceived value brought by luxury brands to consumers. However, the value of quality puts aside the luxury brand and returns to the function of the product itself. Previous studies have shown that consumers value the quality and heritage of second-hand luxury goods, which means they pay more attention to the quality of second-hand luxury goods themselves [36]. Tynan et al.’s research shows that the production process and durability of products are important dimensions for consumers to choose products [37]. For successful luxury goods, consumers need to fully realize the high value of luxury goods to make up for the high price [38]. Second-hand luxury goods have stood the test of time and can be resold in the second-hand market, which further proves that second-hand luxury goods are of good quality and worth buying for consumers [14].

Based on the above analysis and research, the perceived value of second-hand luxury goods is closely related to consumers’ purchase intention. Therefore, we propose the following hypothesis:

**H1:** Perceived value has a positive influence on purchase intention.

### Recycling awareness

Previous studies have shown that some scholars define consumers’ recycling of awareness as a trade-off between receiving and giving based on consumers’ environmental aspirations, sustainable expectations, and green needs [39]. As can be seen from the above research, the perceived value of second-hand luxury goods is mainly divided into three dimensions, among which the quality value of second-hand luxury goods often causes consumers to think about environmental protection issues. In the process of making luxury goods, precious materials are used, such as deerskin and crocodile skin. In addition, the production technology of luxury goods is superb and the quality value is high, so it is a waste of resources to sell luxury goods only once and then discard them [40]. Cervellon et al.’s research shows that high-quality luxury goods can further combine second-hand luxury with the concept of sustainability [41]. In addition to the quality value of second-hand luxury goods, their social value will also have an impact on consumers’ awareness of recycling. With the improvement of consumers’ social status, they hope to show their higher awareness of environmental protection by buying second-hand luxury goods, which can also improve their social affinity [42,43]. Therefore, through the sale of second-hand luxury goods, the relationship between the perceived value of goods and the awareness of recycling has been enhanced. Therefore, we propose the following hypothesis:

**H2a:** Perceived value has a positive influence on recycling awareness.

According to the analysis above, the quality value of second-hand luxury goods is still high. For example, second-hand luxury bags will not affect the performance of the product itself and can be resold as second-hand products to prove the good quality of the product, so second-hand luxury goods will be regarded as more cost-effective products. Kapferer et al.’s study points out that new luxury goods are often considered unnecessarily expensive, and precious raw materials are consumed in the process of producing new luxury goods, which is also a waste of resources if the goods are only used once [44]. Therefore, consumers with a higher awareness of recycling believe that second-hand luxury goods have a higher price-performance ratio [45]. Therefore, we propose the following hypothesis:

**H2b:** Recycling awareness has a positive influence on the price-performance ratio.

Osburg et al. argue that second-hand luxury goods can be considered sustainable [46]. Because the resale of second-hand luxury goods saves precious raw materials for manufacturing luxury goods, and the subsequent use of second-hand luxury goods can be regarded as a strategy to prevent excessive consumption [13]. Cervellon et al. found that Italian and French consumers would feel guilty after buying expensive new products or wearing fur coats. However, they felt much less guilty when they bought similar second-hand products [41]. Therefore, consumers with a high awareness of recycling are more inclined to choose second-hand luxury goods when buying luxury goods. Therefore, we propose the following hypothesis:

**H2c:** Recycling awareness has a positive influence on purchase intention.

### Price-performance ratio

According to research in the business field, the concept of the price-performance ratio is to buy goods of similar quality with less money. In addition, the price-performance ratio reflects that the performance of goods is worth the high price [15,26,47]. The high quality, social, and emotional value of second-hand luxury goods would drive consumers to buy them, even if the price is not much different from that of new luxury goods. In addition to the direct perceived value of second-hand luxury goods, some consumers believe that buying second-hand luxury goods is an investment in the future because they can be resold after a period of use, which further improves the price-performance ratio of second-hand luxury goods [48]. Therefore, we propose the following hypothesis:

**H3a:** Perceived value has a positive influence on the price-performance ratio.

The lower price of goods is one of the important factors for people to choose second-hand products, which greatly affects people’s purchasing attitude [26]. But from a practical standpoint, luxury goods appeal not only to higher-income consumers who can afford them but also to lower-income consumers who cannot afford such expensive products. Therefore, the lower price of goods has become one of the important reasons for people to choose second-hand luxury goods, which is also one of the reasons for the long-term operation of the second-hand luxury market. This has led to a shift in the attitude of thrifty people who are also luxury aspirants toward second-hand luxury goods, which they no longer see as unpurchasable. Therefore, we propose the following hypothesis:

**H3b:** Price-performance ratio influences consumers’ attitudes positively.

The high price is one of the important characteristics of luxury goods. Since second-hand luxury goods have the characteristics of both second-hand and luxury products, the price is lower than brand-new goods. This means that the affordability of second-hand luxury goods provides consumers with the opportunity to possess luxury goods [49,50], greatly improving consumers’ willingness to buy second-hand luxury goods. On the other hand, when they belong to specific collectibles, the price-performance ratio can be significantly improved [51]. For example, the price of antiques may exceed that of modern luxury goods, so we propose the following hypothesis:

**H3c:** Price-performance ratio has a positive influence on purchase intention.

### Attitude

Consumers’ attitude indicates their evaluation of the behavior of buying second-hand luxury goods. Qin’s study found that consumers’ evaluation of second-hand luxury goods depends on the value of the products themselves [52]. When consumers feel that the value of the products is consistent with their image, it is more likely for them to buy such products [53]. Luxury brands will bring inner satisfaction to consumers and improve their social status at the same time. Therefore, we propose the following hypothesis:

**H4a:** Perceived value has a positive influence on attitude.

Attitudes are sets of beliefs about a certain object or an act, which may translate into the intention to carry out the act. The expression of intention is the determination to act in a certain way, and the attitude-behavior correlation is greater when this determination is operated as a specific environmental behavior rather than a general attitude toward the environment [52]. Jain et al.’s study found supporting evidence for the influence of attitude on the purchase intention of luxury goods [54]. Consequently, if consumers recognize the behavior of buying second-hand luxury goods in their hearts, they show a positive attitude towards second-hand luxury goods and are willing to buy them. In addition, consumers’ attitudes toward the social functions of luxury brands also show a positive relationship with consumers’ purchase intentions [15]. Wilcox et al.’s research shows that the social functional attitude here mainly refers to self-expression attitude and self-presentation attitude, and they tend to improve their external image by improving their consumption level [55]. Therefore, we propose the following hypothesis:

**H4b:** Attitude influences purchase intention positively.

### Subjective norms

The theory of planned behavior (TPB) assumes that behavioral intention consists of subjective norms, attitudes, and perceived behavioral control. In this context, subjective norms are a set of social factors that reflect perceived social pressure generally from colleagues or family members to carry out the behavior [20]. Zhang et al.’s research finds that, especially under the influence of Chinese collectivist culture, social recognition is particularly important, so consumers will buy second-hand luxury goods to improve their social recognition [56]. Under the influence of the current social background, the consumption of luxury goods is mainly to conform to the mainstream social trend and satisfy the desire for social recognition [57]. Arora et al.’s research points out that luxury consumption is considered a kind of social status consumption [58,59], so the consumers are willing to bear the economic pressure to buy luxury goods. When consumers buy second-hand luxury goods, they will be recognized by the people around them, and the recognition of the people around them makes consumers more and more believe that buying second-hand luxury goods is the correct behavior. Objectively speaking, what people say around them may not necessarily reflect their true opinions. However, as luxury brands have always been regarded as the representatives of fashion trends and social status, such normative influence will encourage people around to praise them blindly, thus stimulating consumers to buy second-hand luxury goods. Therefore, we propose the following hypothesis:

**H5:** Subjective norms have a positive influence on purchase intention.

### Perceived behavioral control

Perceived behavioral control is defined as the perception of how easy or difficult it is to perform a particular behavior based on one’s experience and expected related barriers [20]. The perceived behavior of buying second-hand luxury goods can represent whether the purchasing power and some other resources like related knowledge of consumers are sufficient to support their purchase of such products [60]. For example, consumers need to have enough income to buy second-hand luxury goods, and they also need to know some reliable channels to buy them instead of some imitations. In the context of digitization and the wide use of social media, the number of second-hand products trading is increasing [61], and the number of second-hand luxury retailers in the market is also gradually increasing. In addition, under the current economic background, the proportion of low-income groups is gradually expanding due to the difficulty in employment, but this does not mean that they will not choose luxury goods. As mentioned above, luxury goods appeal not only to higher-income consumers who can afford them but also to lower-income consumers who cannot afford such products and consumers would buy second-hand luxuries at lower prices. Studies have shown that the increase in personal income does have a positive impact on the purchase of luxury goods, but it is not a necessary condition, especially for the young [62]. The relatively low price of second-hand luxuries provides opportunities for low-income consumers to have access to luxuries. Therefore, we propose the following hypothesis:

**H6:** Perceived behavioral control influences purchase intention positively.

**Fig 2:**
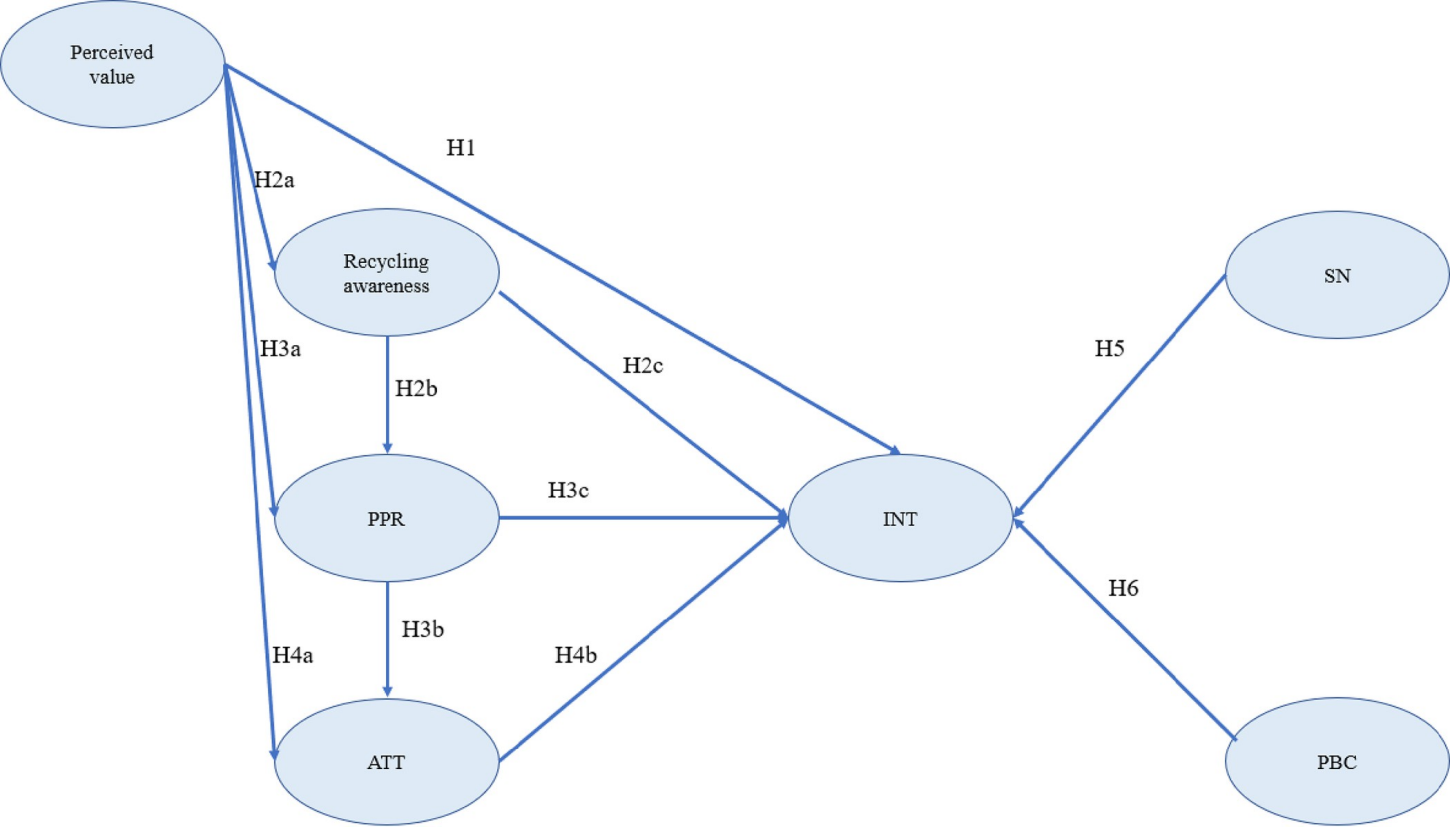
The framework of the study.

To sum up, existing research conclusions prove that factors affecting consumers’ willingness to buy second-hand luxury goods do not work in isolation, which means that the interrelationship between constructs should also be taken into consideration, so the model constructed in this study is shown in Fig 2.

## Method

### Questionnaire design

This questionnaire is divided into two parts: (1) the first part is the statistics of demographic parameters; (2) the second part is a questionnaire designed for factors affecting people’s satisfaction in purchasing second-hand luxury goods. The second part of the question design contains seven dimensions including attitude (ATT), perceived value (PV), recycling awareness (RE), price-performance ratio (PPR), subjective norms (SN), and perceived behavioral control (PBC), and consumer purchase intention (INT). Among them, the perceived value of consumers to second-hand luxury goods is expanded from three aspects: social value (SV), emotional value (EV), and quality value (QV). The questionnaire was initially integrated into English and then translated into Chinese. After proper adjustment, it would be compared with the original questionnaire, and modified if required. The factors mentioned in this study that influence consumers’ purchase of second-hand luxury goods are all adapted from previous studies, so the content of the questionnaire is considered authoritative. The scales of environmental awareness, price-performance ratio, purchase intention, and perceived value of goods are adapted from the study of Lou et al. [14]. The attitude scale is adapted from the study of Tu et al.’s, which intended to reflect the attitudes of consumers by surveying their usage of second-hand luxury goods after purchase [24]. The questionnaire on perceived behavioral control was adapted from Stolz’s study which tested factors that discourage consumers from buying second-hand luxury goods [51]. Finally, the questionnaire of subjective norms is adapted from the study of Li et al. and is designed to explore the influence of people around and the social environment on consumers’ purchase of second-hand luxury goods [20]. The details of the questionnaire are shown in Table 1. In the analysis of the questionnaire, all questions are set into the Likert five-point scale for measurement, which is widely used in the field of social science, with 1 indicating strongly disagree and 5 indicating strongly agree.

**Table 1:** Constructs and measurement items.

Constructs		Measurement items
Perceived Value (PV)	Social Value (SV)	SV1: Purchasing second-hand luxury goods can give me social approval
		SV2: Second-hand luxury goods would leave a good impression on other people
		SV3: Second-hand luxury goods would improve the way I am perceived by others
		SV4: Purchasing second-hand luxury goods can improve my image
		SV5: Second-hand luxury goods would enhance my status
	Emotional Value (EV)	EV1: Purchasing second-hand luxury goods makes me feel good
		EV2: I feel like a treasure hunter when buying second-hand luxury goods
		EV3: I feel happy when I can find a second-hand luxury good that corresponds with my style
		EV4: I feel happy when I can find a second-hand luxury good that is not found in mainstream stores
	Quality Value (QV)	QV1: The overall quality of second-hand luxury goods is excellent
		QV2: The quality of second-hand luxury goods is a major reason for buying them
		QV3: Second-hand luxury goods have excellent style and beautiful colors
		QV4: Second-hand luxury goods that meet my quality standards will enter into my purchasing considerations
Attitude (ATT)		ATT1: I will purchase second-hand luxury goods and resell them in the future as a way of investment.
		ATT2: I will purchase second-hand luxury goods for my private collection.
		ATT3: I purchase second-hand luxury goods because I want to know more about luxury goods and want to take this as an entryway to purchase brand-new luxury goods in the future.
		ATT4: I will purchase second-hand luxury goods for personal use.
		ATT5: I will purchase second-hand luxury goods and give them as gifts to others.
Recycling Awareness (RE)		RE1: Buying second-hand luxury helps save resources
		RE2: Second-hand luxury has a positive impact on the environment in that it extends the life of used materials
		RE3: Second-hand luxury goods have more environmental benefits than new luxury products
		RE4: By buying second-hand luxury goods, I feel I am helping to fight against waste
Constructs		Measurement items
		RE5: I enjoy buying second-hand luxury goods because I don’t like objects being thrown away that can still be of use
Price-performance Ratio (PPR)		PPR1: I think second-hand luxury goods are better at the price-performance ratio.
		PPR2: I can afford more things because I pay less when purchasing second-hand luxury goods
		PPR3: I can have more things for the same amount of money if I buy second-hand luxury goods
		PPR4: I feel that I am paying a fair price by purchasing second-hand luxury goods.
Subjective Norms (SN)		SN1: People important to me feel that I should buy luxury goods.
		SN2: People I know buy luxury goods.
		SN3: People I know think it is important to buy luxury goods.
		SN4: Those who are influential on what I do and think recommend my buying luxury goods.
		SN5: The majority of people who are important to me will help me purchase luxury goods.
		SN6: I feel social pressure to buy luxury goods.
Perceived Behavioral Control (PBC)		PBC1: I would be able to buy second-hand luxury products.
		PBC2: I have the resources to buy second-hand luxury products.
		PBC3: I know how to buy second-hand luxury products.
		PBC4: I know how to buy second-hand luxury products.
Purchase Intention (INT)		PI1: I intend to buy luxury goods.
		PI2: I recommend that others buy luxury goods.
		PI3: I will try to purchase luxury goods in the forthcoming months.
		PI4: I would purchase luxury goods if I could find them easily.

### Sample and data collection

The research protocol in this study was approved by the Ethics Committees of Sichuan Normal University. Since there is a wide range of potential consumers to purchase second-hand luxuries, there are no specific requirements for the respondents of this questionnaire. Finally, 567 questionnaires were collected using the Questionnaire Star system in this study from October 2022 to December 2022, and all the participants signed the written informed consent. There is a wide range of people participating in the questionnaire without significant regional concentration.

The participants’ demographic profiles are listed in Table 2. According to the sample of this survey, the proportion of male (54.5%) and female (45.5%) respondents was almost equal. From the perspective of age, most of the respondents were aged between 18 and 65, with 13.4% below 18, 77.1% between 18 and 65, and 9.5% above 65. In terms of educational attainment, the proportion of participants with a bachelor’s degree was comparable to that with a high school degree. Those with a bachelor’s degree accounted for 47.6 percent, followed by those with a high school degree (34.2 percent). Twelve percent of the participants had a junior high school education or below, and the remaining 6.2 percent had a master’s degree or above. There were differences in monthly income levels among the respondents. About 15.9% of respondents have a monthly income of less than 2000, and 32.9% have a monthly income in the range of 2,000–5,000. Respondents with a monthly income of 5,000–7,000 account for 40.4% while the rest have a monthly income of more than 7,000, accounting for 10.8%.

**Table 2:** Demographic data for respondents.

Demographic category	Item	Subjects	
		Frequency	Percentage/%
Gender	Male	309	54.5
	Female	258	45.5
Age	0–18	76	13.4
	18–65	437	77.1
	≥65	54	9.5
Education	Junior school	68	12
	Senior school	194	34.2
	Undergraduate	270	47.6
	Graduate student	35	6.2
Income	≤2000	90	15.9
	2000–5000	187	32.9
	5000–7000	229	40.4
	≥7000	61	10.8

### Data analysis

SPSS Version 24 for Windows was adopted to obtain the frequency distributions of each item. Partial Least Squares Structural Equation Model (PLS-SEM) is a second-generation structural equation modeling technique that employs a component-based approach, and an alternative and complementary technique to the covariance-based Structural Equation Model (CB-SEM). Compared with covariance-based approaches, minimal restrictions on measurement scales, sample size, and data distributions were placed on the PLS-SEM [63]. The PLS-SEM approach is also suitable for developing new models [64], and, given few previous research on the recycling and price-performance ratio, it was considered more suitable for this study. Another advantage of PLS-SEM is the avoidance of problems with inadmissible solutions and factor indeterminacy encountered by covariance-based approaches [65].

For PLS-SEM, at least ten times as many cases as the larger of (1) the maximum number of measuring items for any construct or (2) the maximum number of links to any construct in the proposed model are required [63], and the sample exceeds this criterion comfortably. Chin recommended that the analysis of PLS models should be conducted in two stages: assessment of the measurement model and evaluation of the structural model [64], which are adopted in this paper.

## Results

### Assessment of the measurement model

To evaluate the validity of the measurement model, it is of necessity to assess construct validity by checking the convergent and divergent validities of the latent variables. Convergent validity requires that the loading of each measurement item on its latent construct exceeds the criterion and is statistically significant [64]. The significance of the loadings was evaluated by the bootstrapping procedure (5000 sub-samples), as recommended by Chin [64], and all the t-values were statistically significant. The minimum acceptable level for the loadings or correlations between the items and the construct, which indicate the individual reflective-item reliability, is 0.70 as shown in Table 3 [64], and the loadings for all items in this analysis exceeded this. The average variance extracted (AVE) for each construct, the amount of variance captured by the construct relative to that attributable to measurement error, is listed in Table 3 and above 0.5 in this analysis [63], and therefore all the constructs are convergent.

**Table 3:** Construct validity and reliability.

Construct	Measurement item	Factor loadings	Cronbach’s alpha	Composite reliability (CR)	Average variance extracted (AVE)
Perceived value (PV)*	SV	0.758	0.798	0.836	0.630
	EV	0.861			
	QV	0.758			
Social Value (SV)	SV1	0.853	0.886	0.917	0.688
	SV2	0.848			
	SV3	0.805			
	SV4	0.784			
	SV5	0.853			
Emotional Value (EV)	EV1	0.864	0.876	0.915	0.728
	EV2	0.844			
	EV3	0.867			
	EV4	0.838			
Quality Value (QV)	QV1	0.880	0.878	0.916	0.733
	QV2	0.848			
	QV3	0.832			
	QV4	0.863			
Recycling awareness (RE)	RA1	0.767	0.882	0.914	0.681
	RA2	0.815			
	RA3	0.849			
	RA4	0.850			
	RA5	0.841			
Price-performance Ratio (PPR)	PPR1	0.856	0.855	0.902	0.697
	PPR2	0.804			
	PPR3	0.849			
	PPR4	0.830			
Attitude (ATT)	ATT1	0.836	0.854	0.895	0.631
	ATT2	0.777			
	ATT3	0.757			
	ATT4	0.773			
	ATT5	0.828			
Subjective norm (SN)	SN1	0.784	0.910	0.931	0.692
	SN2	0.854			
	SN3	0.809			
	SN4	0.866			
	SN5	0.809			
	SN6	0.864			
Perceived behavioral control (PBC)	PBC1	0.808	0.855	0.902	0.697
	PBC2	0.845			
	PBC3	0.864			
	PBC4	0.822			
Behavior intention (INT)	INT1	0.857	0.851	0.899	0.691
	INT2	0.812			
	INT3	0.791			
	INT4	0.864			

* Perceived value is a second-order construct that includes three first-order constructs (Social Value, Emotional Value, and Quality Value).

Internal consistency or construct reliability is indicated by the composite reliability (CR) scores and Cronbach’s alpha. The composite reliabilities for the latent constructs range from 0.836 to 0.931 while Cronbach’s alpha is from 0.798 to 0.910, which are shown in Table 3 and higher than the minimum recommended level of 0.7 in the process of model development [64].

Discriminant validity of the measurement model at the indicator level is demonstrated when each measuring item loads more highly on its assigned latent construct in the theoretical model than on other latent variables, which is satisfied in this analysis. In terms of the latent variable level, discriminant validity can be assessed by examining the AVE. The square root of the AVEs for every construct is greater than the correlations between them and other constructs in this study, which is listed in Table 4, and it provides support for the discriminant validity of the latent variables based on the Fornell-Larcker criterion [66]. According to the Heterotrait-Monotrait Ratio (HTMT) criterion and Table 5, the discriminant validity of the latent variables is also valid.

**Table 4:** Discriminant validity based on the Fornell-Larcker criterion.

	ATT	INT	PBC	PPR	RE	SN	VA
ATT	**0.795**						
INT	0.556	**0.831**					
PBC	0.456	0.351	**0.835**				
PPR	0.446	0.417	0.340	**0.835**			
RE	0.576	0.648	0.659	0.562	**0.825**		
SN	0.682	0.564	0.586	0.657	0.589	**0.832**	
VA	0.508	0.591	0.405	0.494	0.509	0.425	**0.794**

**Table 5:** Discrimination validity test results for the constructs (Heterotrait-Monotrait Ratio (HTMT) Criterion:<0.85).

	ATT	INT	PBC	PPR	RE	SN	VA
ATT							
INT	0.833						
PBC	0.761	0.596					
PPR	0.594	0.656	0.582				
RE	0.436	0.776	0.487	0.690			
SN	0.678	0.682	0.804	0.471	0.589		
VA	0.455	0.687	0.502	0.588	0.788	0.791	

### Assessment of the structural model

The predictive accuracy of the constructs in the structural model was evaluated by the determination coefficient R^2^, and Falk and Miller recommend a minimum acceptable value of 0.10 for R^2^. The R^2^ values of the constructs in the proposed model are greater than the minimum value (RE R^2^ = 0.371; PPR R^2^ = 0.412; ATT R^2^ = 0.601; INT R^2^ = 0.764), which indicates that the predictive accuracy of endogenous constructs is satisfied in this study. Besides, the cross-validated redundancy index Q^2^ is adopted to evaluate the predictive relevance of the structural model. The Q^2^ of the endogenous constructs in this study was above 0, which indicates the satisfied predictive relevance of the structural model.

The structural path model was evaluated by assessing the value and statistical significance of the path coefficients. The statistical significance of the paths can be evaluated by the t-values and p-values obtained by bootstrapping while re-samples of 5000 are generally supposed to provide reasonable standard error estimates [63]. Table 6 lists the hypothesis test results, and all the hypotheses are supported except for H5. It can be indicated that PV (β = 0.368, p = 0.000), RE (β = 0.098, p = 0.007), PPR (β = 0.064, p = 0.017), ATT (β = 0.175, p = 0.003), and PBC (β = 0.154, p = 0.010) are positively associated with the purchase intention except for SN (β = 0.142, p = 0.106), indicating that H5 is statistically rejected. Apart from the purchase intention, PV is positively related to RE (β = 0.609, p = 0.000), PPR (β = 0.433, p = 0.000), and ATT (β = 0.655 p = 0.000). What’s more, RE is positively associated with PPR (β = 0.187, p = 0.000) while PPR is positively associated with ATT (β = 0.171, p = 0.004).

**Table 6:** Hypothesis test results.

Hypothesis		Path	Path coefficient	Standard Deviation	T Statistic	P Value*	Comment
H1							
		PV -> INT	0.368	0.087	4.212	0.000	Supported
H2							
	H2a	PV -> RE	0.609	0.016	38.062	0.000	Supported
	H2b	RE -> PPR	0.187	0.036	5.078	0.000	Supported
	H2c	RE -> INT	0.098	0.036	2.690	0.007	Supported
H3							
	H3a	PV -> PPR	0.433	0.039	10.972	0.000	Supported
	H3b	PPR -> ATT	0.171	0.060	2.848	0.004	Supported
	H3c	PPR -> INT	0.064	0.027	2.381	0.017	Supported
H4							
	H4a	PV -> ATT	0.655	0.057	11.491	0.000	Supported
	H4b	ATT -> INT	0.175	0.059	2.945	0.003	Supported
H5							
		SN -> INT	0.142	0.088	1.617	0.106	Rejected
H6							
		PBC -> INT	0.154	0.060	2.577	0.010	Supported

* The bootstrap t-value was obtained using 5000 subsamples [1,2].

[1] J.F.J. Hair, H.G.T. M., C. Ringle, M. Sarstedt, A primer on partial least squares structural equation modeling (PLS-SEM), Sage publications2016.

[2] J.F.J. Hair, S. M., C.M. Ringle, S.P. Gudergan, Advanced issues in partial least squares structural equation modeling, Sage Publications2017.

## Discussion

It is found that H1 is valid, which means that the perceived value of second-hand luxury goods has a positive impact on consumers’ purchase intention. As hypothesized, consumers believe that the social value of second-hand luxury goods can play a role in showing their social status. This is consistent with the conclusion obtained from Kessous’s study [67]. All these studies agree that consumers use products from luxury brands to create a good image and seek a positive public reflection on themselves. Therefore, this study validates the significance of the social value of second-hand luxury goods and finds that consumers tend to choose products that can create and maintain the ideal self-image they show to the public. In addition to improving one’s social status, the basic functions of the product are equally important so the high-quality value is also one of the reasons consumers choose second-hand luxury goods. This is consistent with Roberts’s research, which also believes that second-hand luxury goods with high quality value can still be used for a long time [68]. According to the questionnaire survey, about 80% of respondents think that second-hand luxury goods have excellent color and style, and about 86% of respondents think that second-hand luxury goods are of good quality. The data obtained in this part came from the respondents who have chosen 4 and 5 in the questionnaire, so consumers believe that second-hand luxury goods are worth buying in terms of both appearance and quality. The emotional value provided by second-hand luxury goods to consumers is also one of the reasons to encourage consumers to choose second-hand luxury goods, which is consistent with the conclusion of Farhad Aliyev et al.’s research [69]. Since scarcity is one of the characteristics of second-hand luxury goods, many luxury goods can only be obtained through the second-hand market due to the limited number of them [70]. Research has shown that consumers enjoy the experience of searching a range of products for used luxury items they need [71]. This process is very enjoyable for the consumer and regarded as a kind of "treasure hunter" experience. Given the need for uniqueness among contemporary young people, they will use this item as a way to express their identity when they find second-hand luxury goods that are different from brand new ones on the market. This emotional value will make the shopping experience of consumers enjoyable, and thus enhance their willingness to buy second-hand luxury goods.

The analysis of H2a shows that the perceived value of second-hand luxury goods has a positive impact on consumers’ awareness of recycling. Similarly, the analysis of H3a shows that the perceived value of goods has a positive impact on the price-performance ratio of goods. This is the same conclusion reached by Athwal et al.’s study [72]. The study found that the high perceived value of second-hand luxury goods with lower prices will make consumers think that the products they buy have a higher price-performance ratio. In addition, second-hand products are conducive to the recycling of resources, and buying second-hand luxury goods is also considered a kind of environmental protection. Therefore, in the era of advocating the importance of environmental protection, consumers will start to think about whether their behavior is conducive to environmental protection, and purchasing second-hand luxuries may be an option for these consumers. Through data analysis, it can be seen that H2b is established. Ramayah et al.’s study found that consumers with a high awareness of recycling would think that the second-hand luxury goods they bought have a higher price-performance ratio [39], which is consistent with the conclusion of this study. The reason is that these people think that they not only get the value of the commodity itself but also fulfill the responsibility of protecting the environment. Therefore, they think that their behavior of buying second-hand luxury goods is endowed with special significance for saving precious natural resources like the fur of rare animals and reducing environmental pollution due to the production process. For consumers with a strong awareness of recycling, their behavior of buying second-hand luxury goods is not only to pay for the products but also to pay for their environmental responsibility. The establishment of H2c indicates that consumers’ awareness of recycling will have a positive impact on the purchase intention of second-hand luxury goods, which is the same conclusion as Athwal et al.’s study [72]. Second-hand luxury goods extend the service life of products by reselling them, thus reducing the consumption of precious resources. Consequently, consumers with a high awareness of recycling will realize this problem and choose second-hand luxury goods for their psychological needs of protecting the environment and saving resources. In addition, it is necessary to analyze the profound motivation underlying the phenomenon. When consumers have a certain social status and have the economic capacity to purchase brand new luxuries, they still choose to buy second-hand luxury goods because they want to establish a social image of high environmental awareness for themselves. In this way, using second-hand luxury goods will not only improve their social status but also help them leave a good social image on the people around them.

The establishment of H3b indicates that the high-price-performance ratio of goods has a positive impact on consumers’ attitudes. According to the questionnaire survey, about 84% of respondents believe that the price-performance ratio of second-hand luxury goods is better, and this data comes from the proportion of respondents who have chosen 4 and 5 in the questionnaire. Stolz’s study reached the same conclusion, and pointed out that the price-performance ratio of a product has a great impact on the consumers’ attitude [51]. The price-performance ratio can be understood from two aspects: first, it can be bought at a lower price when the product performance is similar; second, the performance and function of the product are worth a high price for it. Turunen et al.’s research has confirmed the important role of commodity discounts in second-hand products, and consumers mostly choose second-hand luxuries for the similar reason [48]. As for luxury goods, many products are produced in limited quantity and have a high collection value, so a relatively small number of people own such products, which are easy to sell when the products are first released. Therefore, such limited-edition goods with high collection value can only be bought in the second-hand market after their first release. In this case, the price of second-hand luxury goods may not be lower, but considering the value of the goods, consumers are willing to pay the high bill. Therefore, no matter from any point of view, the high price-performance ratio of second-hand luxury goods will make consumers show a positive attitude towards them. The establishment of H3c indicates that the price-performance ratio of the product has a positive impact on consumers’ purchase intention, and Guzzentti et al. also put forward the same conclusion [73]. They pointed out that commodity price and value are the important factors that affect whether consumers buy or not. Taking these two aspects into consideration, it is the cost performance ratio of the commodity. The higher the cost performance ratio of the commodity, the greater the probability that consumers will buy such products. As mentioned above, luxury goods are often considered to be unnecessarily expensive. According to the results in this study, about 83% of respondents believe that they pay a fair price by buying second-hand luxury goods. For example, some consumers find it difficult to accept a very high price for a luxury bag, but the relatively low price of second-hand luxury goods will make consumers think that the price is more appropriate and increase their willingness to buy second-hand luxury goods. From the analysis above, it can be seen that luxury brands are for the whole society, not limited to high-income groups. However, middle- and low-income people need to consider the price and performance of the goods rather than only focus on the social value brought by luxury goods. Therefore, the high price-performance ratio of second-hand luxury goods will make consumers feel that this product is worth buying, thus increasing consumers’ purchase intention.

Through the verification of H4b, it is found that this hypothesis is valid, which means consumers’ attitudes towards second-hand luxury goods will have a positive impact on consumers’ purchase intention. Schade et al.’s research shows that if the consumer’s attitude towards a certain behavior changes, the consumer’s behavior will also be very different from the past [74]. Kumar et al.’s study also comes to the same conclusion, which shows that consumers will decide whether to buy second-hand luxury goods based on their attitudes [75]. The attitude here is not only the attitude toward products but also the attitude toward luxury brands. Therefore, it can be concluded that a positive attitude will encourage consumers to buy second-hand luxury goods. In addition, when consumers believe that luxury brands can help them define themselves and gain social recognition, they will show a positive attitude toward buying second-hand luxury goods. This positive attitude mainly comes from the perceived value of second-hand luxury goods. It is verified that the perceived value of second-hand luxury goods in H4a has a positive impact on consumers’ attitudes, which can well explain the phenomenon that people are optimistic about second-hand luxury goods. This is the same as the conclusion of Li et al.’s study [20], which indicated that although some consumers are reluctant to accept second-hand products, second-hand luxury goods are different from ordinary second-hand products because of their high perceived value. Moreover, in China, consumers tend to buy luxury goods as a method to maintain their wealth and convey their ideal social image through the social value of luxury brands [76]. Therefore, the perceived value of second-hand luxury goods is worth paying for by consumers, so consumers would have a positive attitude toward them and are more likely to purchase them.

According to the data analysis of this study, H5 is not valid, that is, the research results do not support the positive impact of consumers’ subjective norms on the purchase intention of second-hand luxury goods. Jain’s study believes that the purchase of second-hand luxury goods can be associated with the promotion of social status [77]. Besides, Salem et al. believe that buying second-hand luxury goods is due to the high sensitivity to social norms, so it is concluded that subjective norms will have a positive impact on purchase intention, which is contrary to the conclusion of this study [78]. In this study, the subjective norms are that consumers decide whether to buy second-hand luxury goods according to the evaluation of the people around them. Through the analysis of the questionnaire, it can be seen that the income level of the respondents is mostly below 7000 *yuan*, and their income is not high. Even though consumers would be influenced by subjective norms to some extent, subjective norms are not a determinant of consumers’ purchase intentions because the people around them may not have luxuries and social pressure does not exist.

The establishment of H6 indicates that consumers’ perceived behavioral control has a positive impact on the purchase of second-hand luxury goods. Consumers judge the difficulty of buying second-hand luxury goods based on their cognitive social status and existing resources. With the development of science and technology, the use of social platforms and e-commerce platforms provides a series of platforms for second-hand transactions. Through the analysis of the questionnaire, it can be seen that most interviewees think they have appropriate ways to buy second-hand luxury goods, which proves that the existing resources of most consumers can meet their desire to buy second-hand luxury goods. In addition, with the gradual expansion of the second-hand luxury market, the publicity will be intensified, so the difficulty of consumers buying second-hand luxury goods will be further reduced. The study of Jain et al. combined with the analysis of TPB theory and came to the same conclusion [54]. This study believes that under the premise of sufficient resources, consumers will pay attention to the luxury brands used by their idols to determine their purchase goals, which is also one of the embodiments of high perceived behavioral control.

## Implications

Most existing studies focus on the direct impact of certain factors on consumption and the intention to buy second-hand luxury goods, which means that past studies have ignored the interrelationship between the factors to some extent. To sum up, in the face of the complex purchasing psychology of consumers and the dynamic luxury consumption market, it is difficult to draw an accurate conclusion without in-depth analysis, and inaccurate conclusions may even limit the understanding of luxury consumption.

To make up for these deficiencies, the structural equation model was used in this study to conduct an in-depth analysis of the factors affecting the purchase intention of second-hand luxury goods. The results of data analysis show that all hypotheses are valid except that subjective norms have no significant effect on consumers’ willingness to buy second-hand luxury goods. Considering the recycling awareness of consumers, the research in the commercial field and environmental protection field can be combined to protect the environment while developing the economy, which is in line with the current concept of sustainable development. Therefore, this study focuses on analyzing the impact of consumers’ recycling awareness on their willingness to buy second-hand luxury goods. It is well known that many luxury goods are made from the fur of endangered or rare animals, and to ensure the quality of the products, the consumption of precious raw materials in the production process is much higher than that of ordinary commodities. For example, the raw material for some bags is a crocodile belly, and one crocodile belly is not enough to make a bag, so it may take several crocodile bellies to make a luxury bag. According to the current international situation, biodiversity needs to be maintained by all, and protecting endangered animals is the common responsibility of all mankind. Once many creatures become extinct, humans will never see them again, which is a great loss to nature. Although today’s new luxury products are also developing in a green and sustainable direction [79], their production still needs to consume a lot of precious raw materials. Therefore, studying this can help consumers realize the environmental benefits induced by buying second-hand luxury goods, and help improve consumers’ environmental awareness.

Through the analysis of the model, it is found that the perceived value of second-hand luxury goods is one of the very important research factors in this study, which affects the purchase intention of consumers as well as other factors. The social value and emotional value of second-hand luxury goods are mostly provided by luxury brands. Therefore, for marketing, it is necessary to attach importance to the quality of goods, strictly control the entry standard of goods into the market, and maintain the high-quality value of second-hand luxury goods. By improving the reputation of second-hand luxury goods, consumers’ doubts about product quality can be eliminated, more people can choose second-hand luxury goods, and economic development can be promoted.

## Limitations

In addition to the content analyzed above, there are still many limitations in this study, but it also provides some directions for future research. In the process of the questionnaire survey, respondents mostly target people with monthly income between 2000 and 7000, which leads to the limitations of the survey group itself. The price of second-hand luxury goods is relatively high, and the income of respondents determines that they may not be able to pay for second-hand luxury goods. Therefore, this is one of the important reasons why subjective norms have no significant influence on consumers’ purchase intention. Therefore, the scope of interviewees can be further expanded in the follow-up research, and different income groups can be taken as research objects for analysis, to more accurately obtain the relationship between subjective norms and consumers’ purchase intention.

## Conclusions

This study mainly explains the main factors that affect consumers’ behavior toward buying second-hand luxury goods. The results show that all the hypotheses proposed in this study are valid except for the subjective norms. As a basic attribute of goods, the perceived value of second-hand luxury goods is of great significance to consumers’ purchase intention, among which the connotation of perceived value is also very rich. Through analysis, this research concludes that social value, emotional value, and quality value under perceived value have positive effects on consumers’ purchase intention. Price-performance ratio, as an important factor for consumers to evaluate whether a product is worth buying, also plays an important role, which is related to the perceived value just mentioned. It is precisely because of the high perceived value of second-hand luxury goods that consumers’ attitudes towards second-hand luxury goods will develop better. Consumers tend to weigh and choose what they think is best worth buying, even if the price is higher, they are willing to pay for the quality of the product. Similarly, consumers’ awareness of recycling will make them feel that what they get from buying second-hand luxury goods is not just a commodity, but more harvest of environmental protection. Therefore, the research finds that consumers’ awareness of recycling will encourage them to choose second-hand luxury goods. As the main content of TPB theory, the control of consumers’ perceived behavior provides an important condition for them to buy second-hand luxury goods. These conclusions can be used as reference materials for future academic research, and can also provide suggestions for the marketing of second-hand luxury goods.

## Supporting information

S1 Appendix Ethics Review Form for Studies at Sichuan Normal University. (DOCX)
