# Supplementary material for: Purchase intention for second-hand luxury goods: An empirical study of Chinese consumers
Source: PLoS One. 2024 Jun 5;19(6):e0304967. doi: 10.1371/journal.pone.0304967 (PMC11152274; doi:10.1371/journal.pone.0304967)
Supplement: S1 Appendix — (DOCX) [file pone.0304967.s001.docx]

| **四川师范大学伦理审查表**  **(Ethics Review Form for Studies at Sichuan Normal University)** |
| --- |
| 1.课题题目(Project Title)  中国消费者购买二手奢侈品意愿研究  (Purchase intention for second-hand luxury goods: An empirical study of Chinese consumers) |
| 2.基本信息(Basic information) |
| 2a.研究负责人(Principle investigator) |
| 姓名(Name): 颜冠峰 (Guanfeng Yan) |
| 单位(Institution):  工学院 (School of Engineering) |
| 电话(Tel): 15882176535 |
| 邮箱(e-mail): yanguanfeng@sicnu.edu.cn |
| 2b.研究参加人员(Participating researchers) |
| 姓名(Name) 单位(Association) 邮箱(e-mail) |
| 1 李艳洁(Yanjie Li) 工学院(School of Engineering) 1211081854@qq.com |
| 2 张天海 (Tianhai Zhang) 工学院(School of Engineering) 410829016@qq.com |
| 3 穆成林 (Chenglin Mu) 工学院(School of Engineering) 1223522981@qq.com |
| 4 |
| 5 |
| 6 |
| 7 |
| 8 |
| 2c.科研基金  基金名称(Name)：国家自然科学基金 (National Natural Science Foundation of China)  基金来源(Source): 国家自然科学基金委员会 (National Natural Science Foundation Board of China)  基金号(No.): No. 41902296  主持人(Principle investigator): 颜冠峰 (Guanfeng Yan) |
| 2d合作单位(Collaboration)  无 (N/A) |
| 3.研究目标(Research aim )  本研究以中国消费者为样本，从消费者心理角度，探讨了与消费者（回收意识、主观规范、态度、感知行为控制）和产品（感知价值、性价比）相关的因素对二手奢侈品购买意愿的影响。  (From the perspective of the psychology of consumers, the influence of factors related to consumers (recycling awareness, subjective norms, attitudes, perceived behavioral control) and products (perceived value, price-performance ratio) on the intention to buy second-hand luxury goods is explored in this study through an online survey with Chinese consumers as a sample.) |
| 4.研究背景及意义(Background & Significance)  二手奢侈品既具有奢侈品的特征，如感知价值，包括社会价值、情感价值和质量价值，也具有二手商品的性价比。扩大二手奢侈品市场对保护环境和节约珍贵的自然资源具有重要意义，因此研究购买意愿的决定因素具有重要意义。  (Second-hand luxury goods feature both characteristics of luxury products like perceived value including social, emotional, and quality value, and second-hand goods like price-performance ratio. Enlarging the second-hand luxury market is of significance to protect the environment and save rare and valuable natural resources, and thus investigating the determinants of purchase intention is meaningful.) |
| 5.初步成果或证据(Preliminary Results & Evidence)  通过调研发现，以下因素可能会影响消费者购买二手奢侈品意图，回收意识、主观规范、态度、感知行为控制，感知价值和性价比。  (Through research, it has been found that the following factors may affect consumers' intention to purchase second-hand luxury goods: recycling awareness, subjective norms, attitudes, perceived behavioral control, perceived value, and cost-effectiveness) |
| 6.实验设计与方法(Experimental design & method) |
| 6a.具体实验设计(Specific methods)  依据前人研究获得相关问卷，利用问卷星将问卷分发给消费者。  (Based on previous research, relevant questionnaires are obtained and distributed to consumers using Questionnaire Star.) |
| 6b.实验时间表(Timeline)  2022.9-2022.11  (Sep 2022 – Nov 2022) |
| 7.受试人(Human subjects) |
| 7a.受试者征集(Recruitment of subjects)  利用问卷星分发问卷。  (The questionnaire will be distributed by Questionnaire Star system) |
| 7b.受试者入选要求(Inclusion criteria)  无特别要求  (No specific requirements) |
| 7c.受试者排除标准(Exclusion criteria)  无特别要求  (No specific requirements) |
| 8.人体安全与相关保护手段 |
| 8a.受试者是否签署同意书(Informed consent)  是  yes |
| 8b.受试者筛选方法(Screen method)  无  (N/A) |
| 8c.隐私与保护措施(Privacy & Confidence Provision)  被试的隐私会得到保护，数据进作为研究使用。  (The privacy of the subjects will be protected and the data will be used for research purposes.) |
| 9.研究负责人的保证书(Certification of Principle Investigator)  本人声明所填写内容属实,并将严格按照申请书中有关内容从事实验和研究。本人并表示严格遵守国家法律和实验室有关规定，同时保护受试人的健康、权益和隐私。本人有责任将实验中出现的问题如实向实验室汇报，并按照实验室学术委员会的要求改正。  My signature below certifies that the research described in this application and supporting materials will be conducted in full compliance with government regulations and laboratory policies, especially those governing human subjects research. I will promptly report any unanticipated problems or adverse events and make prompt corrections upon the recommendations of the scientific committee.  研究负责人签字(Signature of Principle Investigator): 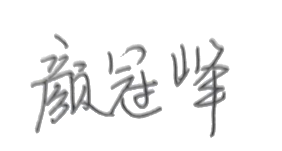  日期(Date):2022.5.28 |
| **以下内容由伦理审查委员会填写(Filled in by the Ethics Review Committee):** |
| 10. 伦理审查委员会意见 (Opinion of the Scientific Review Committee):  同意Agree  伦理审查委员会主席签名(Signature of Chair of Ethics Review Committee): 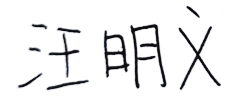  日期(Date): 2022.6.3 |
